# Effect of Crystallinity on the Printability of Poly(ethylene Terephthalate)/Poly(butylene Terephthalate) Blends

**DOI:** 10.3390/polym17020156

**Published:** 2025-01-09

**Authors:** Francesca Aliberti, Maria Oliviero, Raffaele Longo, Liberata Guadagno, Andrea Sorrentino

**Affiliations:** 1 Department of Industrial Engineering, University of Salerno, Via Giovanni Paolo II, 132, 84084 Fisciano, Italy; rlongo@unisa.it; 2 Institute of Polymers, Composites and Biomaterials, National Research Council, P.le E. Fermi, 1, 80055 Portici, Italy; maria.oliviero@cnr.it; 3 Institute of Polymers, Composites and Biomaterials, National Research Council, via Previati n.1/E, 23900 Lecco, Italy; andrea.sorrentino@cnr.it

**Keywords:** additive manufacturing, PET–PBT blends, crystallinity, dimensional stability

## Abstract

This study explores the impact of blending polyethylene terephthalate (PET) with polybutylene terephthalate (PBT) on the thermal, structural, and mechanical properties of 3D-printed materials. Comprehensive analyses, including Fourier-transform infrared spectroscopy (FT-IR), thermogravimetric analysis (TGA), X-ray diffraction (XRD), differential scanning calorimetry (DSC), and mechanical testing, were conducted to assess the influence of blend composition. FT-IR confirmed that PET and PBT blend physically without transesterification, while TGA showed enhanced thermal stability with increasing PET content. XRD revealed that PET and PBT crystallize separately, with the crystallinity decreasing sharply for blends with more than 50% PET. The DSC results indicated that PET effectively slows down the crystallization kinetics of PBT, promoting cold crystallization. Mechanical tests demonstrated that the elastic modulus remains relatively unchanged, but the strain at break decreases with a higher PET content, indicating increased stiffness and reduced ductility. Overall, incorporating PET into PBT improves 3D-printability and dimensional stability, reducing warpage and enhancing print precision, making these blends advantageous for 3D-printing applications.

## 1. Introduction

Extrusion-based Additive Manufacturing (EAM) has emerged for its simplicity, cost-effectiveness, and reliability [1]. By depositing a molten thermoplastic material layer-by-layer according to a CAD model, complex geometries can be created without material waste [2]. Currently, a wide range of materials can be involved in the EAM process, such as PLA (polylactic acid), PETG (polyethylene terephthalate glycol), ABS (acrylonitrile butadiene styrene), ASA (acrylonitrile styrene acrylate), and PA (polyamide) [3]. The technological flexibility of the EAM also allows for the processing of recycled plastics [4], nanocomposites [5], composite [6], and smart materials [7,8]. In this way, EAM responds to the increasing demand for the complexity and multifunctionality of the final 3D-printed product [7]. However, challenges remain, particularly in addressing the requests of complex industrial applications where high mechanical strength at elevated temperatures, barrier properties, and chemical and wear resistance are required [9,10]. Crystallinity is critical for different fields of application such as for the polymeric membranes to retain sufficient mechanical strength in lithium battery applications, for the polymer’s biodegradation and biocompatibility in biomedical applications [9], or, in the case of high-barrier polymers in gas separation membranes, the packaging of healthcare and pharmaceutical goods and chemicals and the housing of fuels (including oxygenated fuels) in tanks and lines in the automotive sector [11]. Semicrystalline thermoplastic polymers such as PP (polypropylene), PE (polyethylene), PEEK (polyether ether ketone), PEN (polyethylene naphthalate), and PBT (polybutylene terephthalate) in the EAM process could be the right solution for many of these applications. However, their use is still limited due to their tendency for thermal shrinkage and warpage, which are more pronounced than in amorphous polymers [12,13]. In fact, during the processing of these materials, severe cooling-induced shrinkage results in the development of large thermomechanical stresses that are relieved by the out-of-plane warpage of the part [14]. In extreme cases, detachment from the build plate, delamination, or part cracking can occur, leading to issues with dimensional accuracy and stability [15]. To address this problem, it is necessary that we highlight the correlation between the EAM parameters and the phenomena that occur during the cooling phase [16]. Many works in literature have tried to model the temperature variation during the EAM process to predict the deformation of the specimen, the mechanical properties, and residual stresses [17,18,19,20]. The effect of the ambient temperature, the printing speed [21], the bed temperature, the layer thickness [12], the part geometry, and the type of toolpath [22] have been considered. Results have shown that it is possible to improve the part quality by optimizing the previous parameters and limiting the increase in both processing time and cost. Another proposed approach to avoid the warpage is based on the incorporation of fillers in pristine polymers such as CaCO_3_ [23], wood powder [24], perlite [23], glass and talcum [25], and natural fibers [26]. These fillers reduce the coefficient of thermal expansion of the polymer matrix and minimize shrinkage. Moreover, in the case of conductive filler, the higher thermal conductivity of composite polymer allows it to converge much faster toward a thermal equilibrium than the neat matrix; consequently, the internal/residual stresses of the material are expected to be reduced [13,27,28]. However, especially at higher concentrations, extrusion issues due to the filler agglomeration can occur [15]. Using fillers with a high aspect ratio, such as carbon fibers, can prevent warp deformations at lower concentrations. However, these fillers significantly increase the anisotropy of the printed and hinder interlayer polymer chain diffusion. Due to all the presented reasons, blending polymers is a promising alternative for reducing warpage and increasing interlayer adhesion [29]. Ho et al. blended PP with 20–40 wt% of elastomeric ethylene-octene copolymers (EOC) to improve the flow properties and interfacial fusion during printing. The authors demonstrated that, owing to its amorphous structure and low melting point, the EOC dispersed phase facilitated the material extrusion process, reduced warpage, and promoted fusion between the printed strands, improving the mechanical properties [30]. Chatham et al. [10] showed that a blend of two polymers with significantly different melting temperatures, like PP and PET, is beneficial for the EAM process since the high melting phase (PET) of the blend start to crystallize at a higher temperature, allowing the low melting phase (PP) to retain molecular mobility until the deposited materials cool down, gaining time to relax internal stresses and minimizing warpage.

Polybutylene terephthalate (PBT) is a semi-crystalline engineering thermoplastic material that has gained commercial interest due to its wide range of applications. Its key features make it an ideal choice in automotive, electrical and electronics, medical, and home appliances [31]. PBT has progressively supplanted nylon materials as the primary option for vehicle electrical connectors as the automotive industry has grown [32]. Reducing defects due to the poor dimensional stability at high temperatures and warp during the sample preparation process would further widen its applicability.

Fundamentally, the chemical structures of PET and PBT are very similar. The main differences involve the ability of the polymer to crystallize under cooling. Under normal processing conditions, the PBT crystallizes efficiently enough to always achieve a high level of organization in its structure. On the opposite, PET, with its more rigid structure, can be obtained both amorphous or semi-crystalline depending on the cooling rate experienced. Contrarily to PET, PBT shows dimensional instability and evident shrinkage, due to the high crystallization rate and crystallinity degree not only when processed by EAM [33] but also in the case of other processes. With high crystallinity, even injection-molded PBT products are easily distorted by anisotropic shrinkage or residual stress [34,35]. This paper aims to explore the potential of blending PBT with PET to enhance the processability of PBT in EAM. Specifically, the study seeks to clarify the impact of the crystallization behavior on warpage and shrinkage in 3D-printed parts, thereby addressing key challenges in the additive manufacturing of semicrystalline polymers.

## 2. Materials and Methods

Polybutylene terephthalate (PBT) was supplied by LANXESS Performance Materials GmbH, Cologne, Germany (Grade: Pocan B1300 000000). It is a high-viscosity PBT resin with a 1.31 g/cm^3^ density. Polyethylene terephthalate (PET) granules were supplied by the Saudi Basic Industries Corporation (Al-Jubail, Saudi Arabia) (SABIC) (Bottle Grade PET BC 212). It is a semi-crystalline amorphous grade with an intrinsic viscosity of 0.84 dL/g. PBT and PET pellets were blended in varying proportions (i.e., 20%, 30%, 50%, 70%, and 80%) using a single-screw extruder (Filament Maker Composer 350 by 3Devo Filament Maker Composer 350, Utrecht, The Netherlands). The extrusion process was conducted with a temperature profile of 230 °C, 250 °C, 240 °C, and 235 °C along the extruder, and the screw was rotated at 5.5 rpm. The resulting spooled filaments, with a diameter of approximately 1.75 mm, were immediately used as input for 3D printing.

The 3D-printing tests were carried out on an Original Prusa i3 MK3S with a 0.4 mm nozzle and a constant layer height of 0.20 mm. The Prusa Slicing Software (Slic3r Prusa Edition 2.8.1) was used to generate the G-code files for printing. Table 1 outlines the key test parameters.

**Figure 1:**
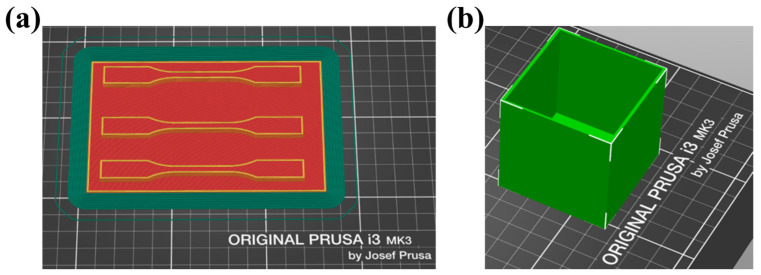
Preview of CAD model of printed samples: (**a**) uniaxial tensile dog-bone specimens; and (**b**) hollow box-shaped specimen.

Uniaxial tensile dog-bone specimens (ISO 527-2-1BA [36]) were printed with their longitudinal axes aligned in the building platform. In this case, various printing conditions were optimized to enhance the adhesion between the first layer and the build platform and prevent any potential distortion. Specifically, the effects of bed adhesive, surface area coverage, and part thickness were studied to establish the optimal printing conditions. A brim design was also introduced to ensure strong adhesion and minimize warping. The brims consisted of two layers of material surrounding the specimens, increasing the surface area in contact with the platform (Figure 1a).

For direct characterization of shrinkage and warpage in the most demanding printing conditions, hollow box-shaped specimens (50 mm side length, 2 mm base thickness, and 40 mm height) were printed with a wall thickness equivalent to a single extruded filament (Figure 1b). In this case, no brim or adhesive was used to increase the adhesion of the part with the building plate.

FT-IR analyses were performed using a Nicolet apparatus (Thermo Fisher Scientific, Waltham, MA, USA) at ambient temperature on extruded filaments of PBT/PET blends. The samples were analyzed in ATR spectra mode from 4000 to 650 cm^−1^, with a wavenumber resolution of 4 cm^−1^ for 64 scans.

The thermal stability of blends in the form of extruded filaments has been investigated using a TGA Q600 (TA Instruments, 159 Lukens Drive, New Castle, DE 19720, USA). Samples were heated from 30 to 800 °C at a heating rate of 10 °C min^−1^ in a nitrogen atmosphere.

A thermal analyzer Mettler DSC 822/400 (Mettler-Toledo, Columbus, OH, USA) equipped with DSC cell purged with nitrogen (50 mL/min) and chilled with liquid nitrogen. Film samples of about 10 mg were subsequently heated, cooled, and reheated at a rate of 10 °C/min from 20 to 280 °C. The glass transition, crystallization, and melting temperature were evaluated from the resulting enthalpic curves.

Wide-angle X-ray diffraction (WAXD) patterns in reflection mode were obtained by an automatic Bruker, Billerica, MA, USA D8 QUEST Advance diffractometer operating at 35 kV and 40 mA (CuKα radiation X-ray source, λ = 0.15418 nm). The increment of theta has been set at 0.0081°, while the time step was 0.200 s. The degree of crystallinity of printed samples was evaluated from X-ray diffraction data, applying the standard procedure of resolving the diffraction pattern into two areas, A_c_ and A_a_, that can be taken as proportional to the crystalline and the amorphous fraction of the polymer, respectively, and calculated, for the 2θ range 10–35°, using the following equation:

$$x_{c}=\frac{A_{c}}{A_{c}+A_{a}}\times 100$$

according to the classical Hermans–Weidinger method [37]. X-ray analysis was performed on PBT/PET blend samples taken from the wall of printed boxes. For comparison, films made with either neat PBT or PET were obtained by, first, hot pressing the relative extruded filaments and, then, cooling them in two different conditions: quenching and slow cooling rate. The quenching procedure consisted of directly putting the melt samples into a bath of liquid nitrogen while the slowly cooled samples were obtained, leaving them after melting via compression molding between the hot plates until the entire setup reached the ambient temperature.

Tensile tests have been performed on printed dog-bone-shaped samples according to the specifics of ISO-527-2-1BA [36], using a Dual-Column Tabletop Testing Systems (INSTRON, series 5967-INSTRON, Norwood, MA, USA) set with a crosshead speed of 1 mm/min. Each stress–strain test was repeated three times.

Dynamic mechanical analysis (DMA) was performed using a DMA 2980 (TA Instruments). Test specimens measuring 40 × 10 mm were cut horizontally (0° direction) and vertically (90° direction) from the box walls to evaluate the morphology of the extruded filaments and bond strength. The samples were subjected to a variable tensile strain, with the displacement amplitude set at 0.1% and the frequency at 1 Hz. The temperature range examined extended from 30 °C to 180 °C, with a constant heating rate of 3 °C per minute.

Microscopic images were taken using a Leica MZ6 (Leica Microsystems, Wetzlar, Germany) stereo microscope equipped with two eyepieces to combine images taken from two different points of view. This gives the final image three-dimensionality and depth. This instrument has a rotating focusing arm for better image visibility and can use polarized lenses. Microscopic images were performed on the printed samples after the break occurred during the tensile mechanical test.

## 3. Results

### 3.1. Characterization of the Extruded Filaments

#### 3.1.1. FT-IR Analysis

An FT-IR analysis was conducted on the extruded filament to examine the possible interaction between PBT and PET after the melt blending process. Several studies in the literature have explored the possibility of transesterification reactions in polyester blends like PET and PBT under specific processing conditions or heat treatment [38,39,40,41,42]. The absence of copolymers due to possible transesterification reactions has been verified via NMR spectroscopy on the printed samples. The NMR results are shown in Figure S1 and commented on in Section S1 of the Supplementary Materials. The ATR spectra have been interpreted in light of the NMR results, which demonstrate the absence of transesterification reactions.

**Figure 2:**
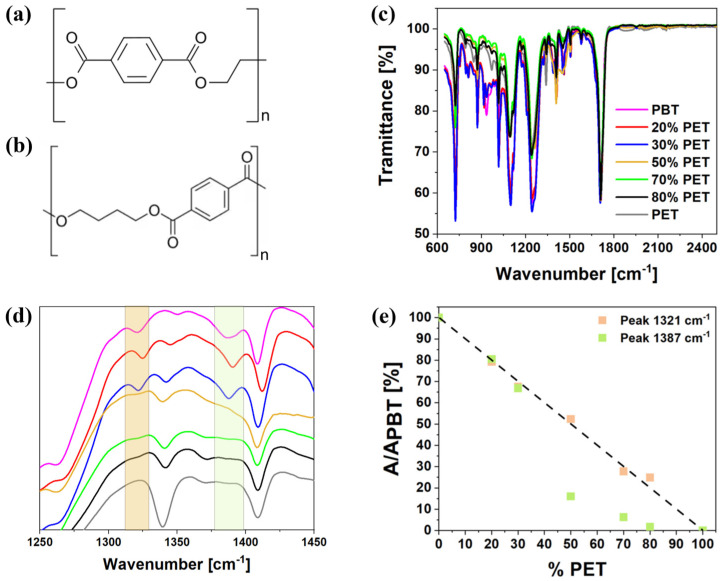
(**a**) Chemical structure of PET; (**b**) chemical structure of PBT; (**c**) ATR spectra of PBT, PET, and their blends; (**d**) enlargement of ATR spectra on the characteristic bands of -CH_2_ groups; and (**e**) ratio between the area of 1321 cm^−1^ and 1387 cm^−1^ peaks of blends and the area of the same peaks of PBT spectrum.

Figure 2c shows the ATR spectra of the characteristic peaks of the pristine PBT [43] and the pristine PET [44] compared with various blends. Given that PBT and PET have similar chemical structures (as shown in Figure 2a,b), with the primary difference being the length of the aliphatic segment between ester groups [31], their ATR spectra exhibit minimal differences in transmission peaks [45,46]. Specifically, PBT contains four CH_2_ groups, while PET has only two. Consequently, the ATR spectra do not show significant differences in terms of transmission peaks. However, two peaks, at 1321 cm^−1^ and 1387 cm^−1^, correspond to CH_2_ twisting vibrations and CH_2_ scissoring and wagging vibrations, respectively [47]. These peaks are characteristic of PBT and are absent in the PET IR spectrum, revealing that the spectra of the blends are a superposition of contributions from the individual components [48]. As seen in Figure 2d, with an increasing PET content, the characteristic PBT peaks at 1321 cm^−1^ and 1387 cm^−1^ gradually disappear.

A quantitative analysis using the deconvolution method shows a good correlation between the area of these peaks and the blend composition (Figure 2e). Specifically, the peak at 1321 cm^−1^ exhibits a linear correlation across all concentrations, while the peak at 1387 cm^−1^ shows a deviation when PET concentration exceeds 50%. This deviation may result from a different structural configuration that inhibits CH_2_ scissoring and wagging vibrations. Moreover, the absence of any significant shifts in peak positions or the appearance of new peaks indicates that no chemical interactions occurred during the extrusion phase. Additionally, no degradation phenomena were observed since the extrusion temperature was kept below the degradation point of the materials.

#### 3.1.2. Thermogravimetric Analysis

**Figure 3:**
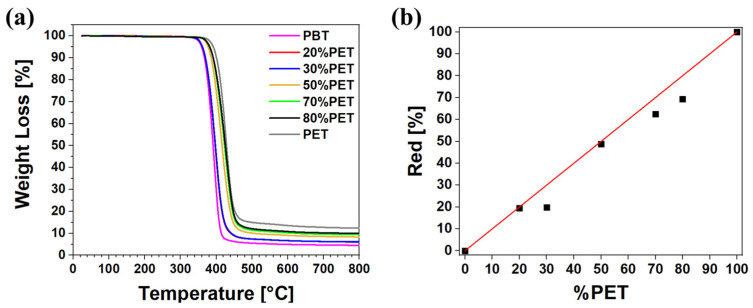
TGA results of PBT, PET, and their blends: (**a**) thermogravimetric curves; and (**b**) relative residue vs. PET content (experimental data (dots) and linear trend (red line)).

The key results of the thermogravimetric analysis, including the maximum degradation temperature (T_max_), the initial degradation temperature corresponding to a 5% weight loss (T_d_), and the residue at 800 °C (R), are summarized in Table 2. As shown in Figure 3, all TGA curves exhibited a single-step degradation process. The TGA analysis confirms that PET has better thermal stability than PBT [49]. Specifically, PET begins to degrade at 392 °C, while PBT starts to lose weight significantly at 359 °C. This difference in thermal stability is likely due to the longer aliphatic segments between the ester groups in PBT chains. The methylene units (-CH_2_-) in PBT represent weaker points in the polymer chain than the segments containing double bonds and aromatic rings. As a result, the increased number of methylene units in PBT, compared to the ethylene groups in PET, facilitates thermal decomposition [50]. Furthermore, the T_d_ values for the PBT/PET blends fall between the initial degradation temperatures of the two pure polymers, with T_d_ increasing as the PET content rises (see Table 2). This trend suggests that PBT and PET are physically compatible after blending, with the two polymers mixing without undergoing any chemical reaction, such as transesterification, as indicated by the NMR results reported in Section S1 of the Supplementary Materials Studies by P.R. Rajakumar et al. [51], and B. Ucpinar Durmaz et al. [49] have shown that transesterification in PBT/PET blends leads to a reduction in thermal stability compared to the original polymers. This reduction is due to the formation of block copolymers in the initial stages and, eventually, random copolymers, which are less thermally stable than the pristine polymers. However, it is well-established that transesterified random copolymers typically form only after prolonged melt mixing at high temperatures [52,53]. Based on the TGA and NMR results, it can be concluded that the PBT/PET blends prepared in this study did not undergo transesterification due to the limited extrusion time and moderate extrusion temperature [52,54].

The TGA curves also reveal the formation of residue or char at higher temperatures. This char, which consists of the organic coking residue, is higher for PET than for PBT [55,56,57]. Specifically, the residue accounts for 4.55 wt% in PBT and 10 wt% in PET. Notably, when considering a relative residue (Red) value, where PBT is zero and PET is 100%, all blends show intermediate values that correlate well with their composition (Figure 3b). This additive behavior further supports the absence of chemical interactions or degradation phenomena between the two polymers. However, a slight deviation from perfect linearity is observed in blends with high PET content (70% and 80%). As found with the FT-IR analysis, this deviation suggests that, in these compositions, some physical interactions between the two polymers might slightly alter the behavior of these blends.

#### 3.1.3. DSC Results

**Figure 4:**
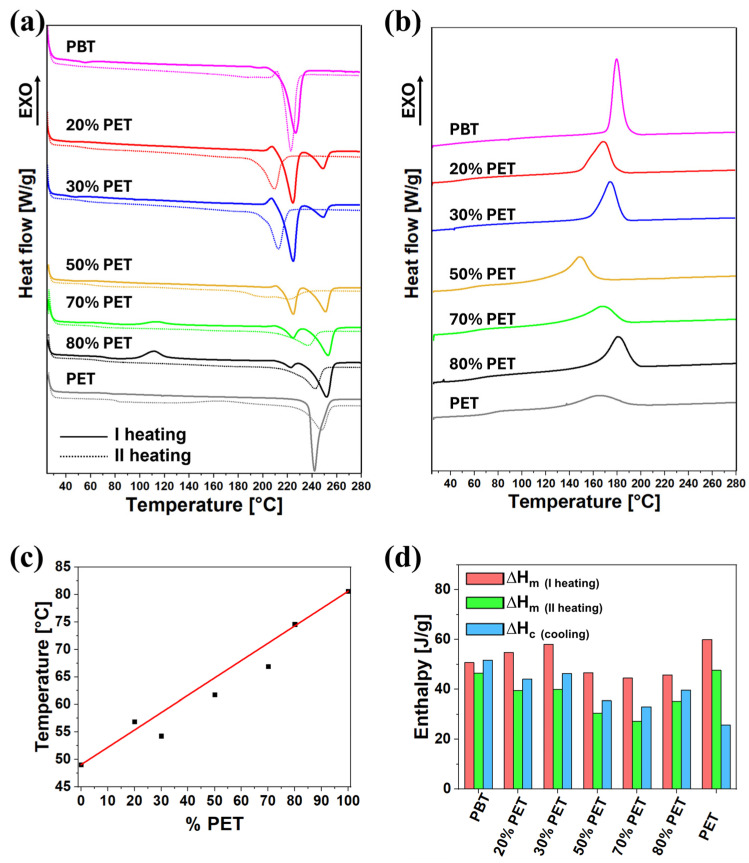
DSC results on spooled filaments: (**a**) first (continuous curves) and second (dotted curves) heating; (**b**) cooling scan; (**c**) Tg values (dots) compared to the mixing rule (red line); and (**d**) melting enthalpy (of both first and second heating) and crystallization enthalpy for all the experimented blends.

Differential scanning calorimetry was carried out of the extruded filament to characterize the crystallization behavior of the blends. Figure 4a,b present the DSC heating and cooling scans for the PET/PBT blends. During the first heating scan (solid curves in Figure 4a), two distinct melting peaks are observed for all blends: one around 225 °C, corresponding to the PBT phase, and another around 250 °C, associated with the PET phase. Interestingly, the melting temperature for PET in the blends is higher than in pure PET, while the melting peak for PBT is lower than that of pure PBT. A detailed explanation of this aspect is given in the Supplementary Materials. Additionally, the 70% and 80% PET blends show a cold crystallization peak during the first heating cycle, indicating that these blends do not crystallize completely during the filament extrusion process. In Section S2 of the Supplementary Materials, the differences emerging from the comparison between the first and the second heating scan of Figure 4a are extensively discussed.

The cooling curves (Figure 4b) reveal a single crystallization peak for all blends in the temperature range between 190 °C and 130 °C. The crystallization temperatures are 165 °C for PET and 181 °C for PBT, while the blends show a crystallization peak temperature lower than pure PBT. This suggests that the miscible PBT phase may act as a nucleating agent for PET, altering its crystallization behavior, as supported by Aravinthan, and Kale [52].

Regarding the glass transition temperature (T_g_), a single T_g_ is observed for each blend, and its value increases as the PET content rises, closely following the linear trend predicted by the rule of mixtures (Figure 4c). This behavior confirms the good miscibility between PET and PBT in the amorphous phase. These findings align with previous DSC studies reported by Avramova [58].

The melting enthalpy (∆H_m_) values, both in the first and second heating scan and the crystallization enthalpy (∆H_c_) during cooling, display a clear trend. For the 50%, 70%, and 80% PET blends, these values are consistently lower than expected, indicating a reduced degree of crystallinity compared to blends with a lower PET content. This trend highlights the impact of PET on the crystallization kinetics and degree of crystallinity of the blends. Numerical values of glass transition temperature (T_g_), crystallization temperature (T_c_), melting enthalpy (∆H_m_) of the first (I) and second (II) heating, and the crystallization enthalpy (∆H_c_) for PET, PBT, and their blends have been summarized in Table 3.

### 3.2. Characterization of the 3D-Printed Samples

#### 3.2.1. Mechanical Properties of the Dog-Bone Specimens

**Figure 5:**
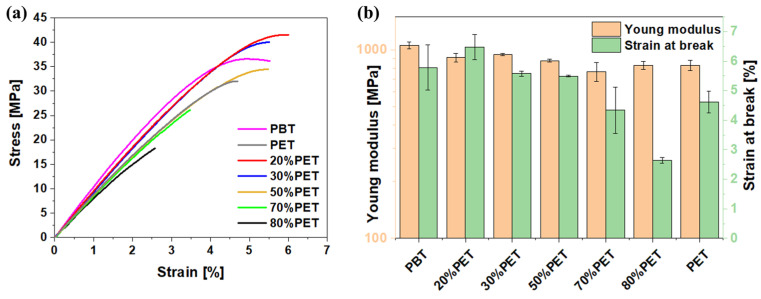
Tensile test results: (**a**) stress–strain curves of PBT, PET, and PBT/PET blends, and (**b**) Young modulus and strain at break variation with PET content.

The impact of the PET concentration on the mechanical properties of printed samples was evaluated through tensile testing at a strain rate of 25 mm/min. Figure 5 presents the representative stress–strain curves for the various blends considered. The average values and distribution of the elastic modulus and strain at break for all blends are summarized in Figure 5b. Specifically, Young’s moduli were derived from the slope of the linear region of the stress–strain response.

The elastic modulus does not show a clear trend with varying PET content, as all blends exhibit similar values, suggesting that the PET concentration has a limited effect. However, the strain at break is significantly affected by the blend composition. Samples with 70% and 80% PET show the lowest strain at break values, indicating increased rigidity. This rigidity likely results from enhanced chain interactions in these samples, potentially due to physical crosslinking such as crystal nuclei or reversible distance-dependent interactions between polymer chains. These properties vary widely with composition due to two competing morphological factors: the deterioration of properties from incompatibility and a resulting two-phase structure and improvement from forming more inter-crystalline structures. Thus, differences in the interaction between blend components can lead to mechanical behavior that deviates from the additivity rule.

**Figure 6:**
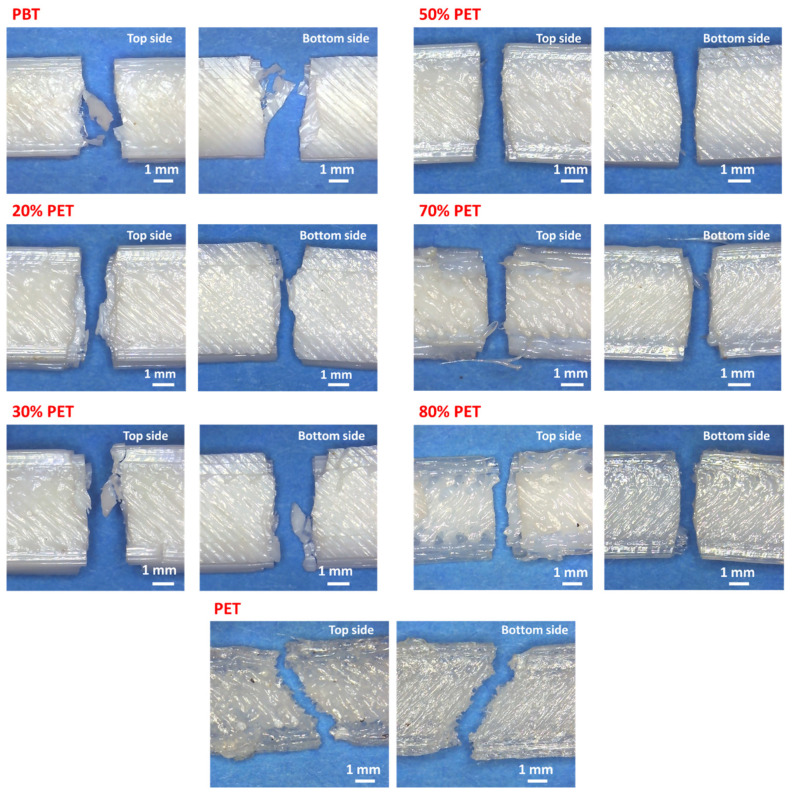
Micrographs of break surfaces for tensile test samples for PBT, PET, and their blends.

Figure 6 shows typical fracture morphologies of the samples, revealing a brittle fracture type for all compositions. The PBT/PET blend surfaces are rough without an observable phase separation, indicating a good compatibility between the polymers. The rough fracture surface suggests that the failure was mainly due to the rupture of deposited filaments rather than interlayer bonds. The quality of interfacial bonding is crucial for the microstructure and mechanical properties of the printed parts. This bonding quality depends on the growth of the neck between adjacent filaments and the molecular diffusion and randomization of polymer chains across the interface. Specimens with a lower PET concentration likely had better adhesion among deposited filaments and layers, displaying improved polymer chain diffusion between layers, resulting in denser parts with smaller interlayer voids.

#### 3.2.2. Morphological Characterization of the Hollow Box-Shaped Specimens

**Figure 7:**
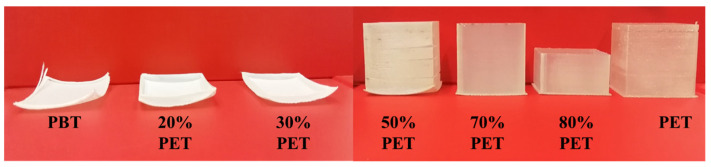
Images of 3D-printed boxes made of different concentrations of PBT/PET blend.

Figure 7 shows photographs of the best-printed samples for each blend. As expected, the PET content significantly influences the morphological characteristics of the samples. Only those with more than 70% PET demonstrated warpage-free and dimensionally stable prints. Blends with less PET content experienced issues with layer adhesion and warpage. Specifically, samples with less than 50% PET could not be fully printed as per the CAD model, due to the severe warpage of the initial layers, causing the detachment from the build plate. Although the box could be fully printed with the 50% PET blend, it still exhibited notable shrinkage, detachment from the build plate, and cracks on the walls. In contrast, the sample with 70% PET showed complete dimensional stability and accuracy.

#### 3.2.3. Thermal Analysis of the Hollow Box-Shaped Specimens

**Figure 8:**
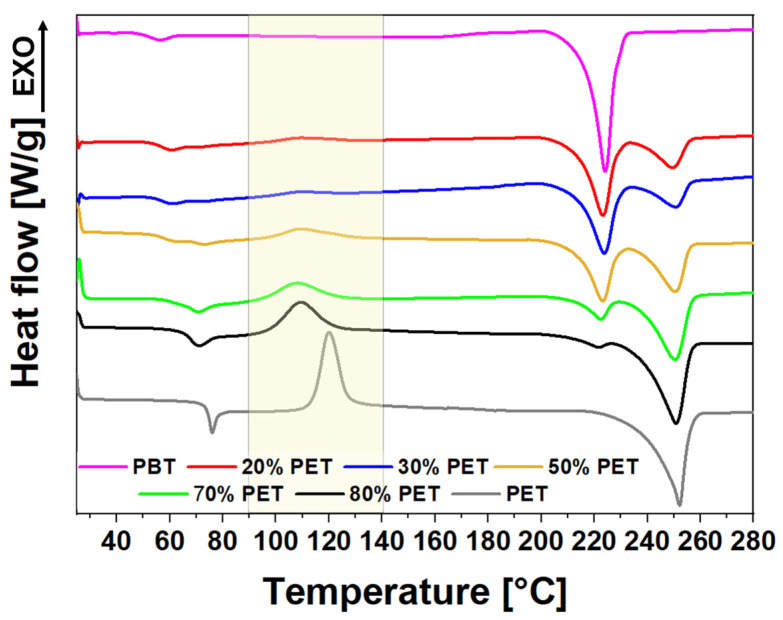
DSC curves of 3D-printed PBT/PET blends.

A DSC analysis was performed to investigate the effect of the PET addition to PBT on the degree of crystallinity of the final blends after the 3D-printing process. Since PBT has an elevated crystallization rate, PET has been added to slow down the crystallization rate during the cooling phase of the 3D-printing process, reducing internal stresses and minimizing warpage. Figure 8 reports the DSC curves obtained on a small piece of 3D-printed box wall for each prepared blend. Moreover, PBT shows a lower T_g_ value (55 °C) than PET, whose T_g_ is 76 °C. For PBT/PET blends, the Tg value tends to increase by increasing the PET content. Regarding the melting region, DSC curves of PBT/PET blends exhibit two melting peaks, each associated with one of the two components of the blends. In fact, the melting peak of PBT at lower temperatures tends to disappear by increasing the PET percentage, while the PET melting peak becomes more evident. This means that PBT/PET blends form separate crystals rather than cocrystals [48,52,58]. For this work, the most interesting region in Figure 8 is the temperature interval between 100 °C and 140 °C where cold crystallization peaks emerge. It can be noted that, by increasing the PET content in the blends, the area of cold crystallization peak tends to increase. This area is proportional to the number of polymer chains that did not crystallize during the cooling phase of the 3D-printing process and then underwent cold crystallization during the heating scan of DSC analysis. Since both PET and PBT are semicrystalline polymers able to crystallize beyond 30% [59], the reason to explain the reduction in crystallinity in the 3D-printed part made of a PBT/PET blend by increasing the PET content is the fact that PET is effectively able to reduce the crystallization kinetic of neat PBT. The cold crystallization peak becomes more evident from the sample composed of 50% of PET, meaning that the crystallinity of 3D-printing PBT/PET blends starts to decrease substantially when the PET percentage exceeds 50%. It confirms the previous results as the increase in PET content in PBT/PET blends leads to an improvement in dimension stability due to a lowering in the degree of crystallinity and, consequently, an increase in the amorphous phase, which causes a small volume reduction during the cooling phase of the 3D-printing process.

DSC curves of printed samples (Figure 8) have been compared to the first heating curves of the DSC analysis performed on the spooled filaments before printing (I heating in Figure 4a) in Figure S2 and commented on in Section S3 of the Supplementary Materials.

#### 3.2.4. X-Ray Diffraction Analysis of the Hollow Box-Shaped Specimens

**Figure 9:**
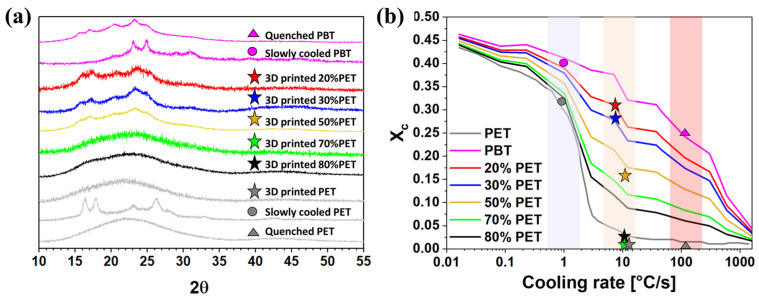
X-ray results: (**a**) X-ray spectra of the 3D-printed sample (stars), of quenched PBT and PET (triangles) and of slowly cooled PBT and PET (circles), and (**b**) comparison between experimental data obtained from graph (**a**) and literature data [59].

Figure 9a reports the X-ray investigation performed on printed blends, and quenched and slowly cooled neat PBT and neat PET. The 3D-printed samples were taken from the wall of the printed boxes for all experimented blends and the neat PET to consider the crystallization phenomenon when the materials exchange heat only with the surrounding air without considering the heat transfer with the heated printing bed. However, for the neat printed PBT, it was not possible to take a piece of the box wall, since, in this case, the 3D-printing process stopped before building the walls. The X-ray spectra of Figure 9a were used to calculate the degree of crystallinity of the samples to compare these results with those obtained by the DSC analysis. By exploiting the deconvolution method, it could be possible to quantify the PET and PBT crystallinity [48,59]. However, it would be quite difficult due to the close positions of the most intense peaks and lie out of the scope of this work whose aim is to understand the effects of blending PET with PBT on the crystallinity degree to improve PBT printability and not to study the crystalline phase of the two polymers in blends. The degree of crystallinity evaluated on the X-ray spectra of Figure 9a according to the procedure described in Section 2 is reported in the form of spot data in the graph of Figure 9b to compare them with the literature data. In fact, in Figure 9b, the continuous lines define the degree of crystallinity of the pristine PBT and PET as well as their blends at different cooling rates taken from Ref. [59]. The circle-shaped points and the triangles represent the crystallinity of the neat PBT and PET obtained in this work under slow and high cooling rates, respectively. According to the experimental conditions in which the samples were obtained, the quenched samples are positioned at a cooling rate of 100 °C/s, while the slow-cooled samples are reported corresponding to about 1 °C/s. The degree of crystallinity of 3D-printed blends is reported in the form of stars at a cooling rate of the order of 10 °C/s. It is worth underlining the differences between pristine PBT and pristine PET. By comparing the X-ray spectra obtained at the two extreme cooling conditions (quenching and slow cooling rate), in the case of PBT, although the contribution of the amorphous phase becomes predominant in the quenched PBT spectrum, some peaks remain, meaning that the cooling rate at which the sample was quenched was not enough to make the PBT amorphous completely. On the other hand, the spectra of the quenched PET and slow-cooled PET are completely different, and it is very evident that the quenching procedure was effective in making the PET amorphous, while the slow-cooled PET is highly crystalline. The fact that, by quenching PBT, it remained semicrystalline while PET became completely amorphous during the same quenching process confirms that these two polymers have two different crystallization rates. The crystallinity of PBT is higher than that of PET over the entire cooling rate conditions investigated. More in detail, between 1 °C/s and 10 °C/s, the neat PET undergoes a rapid change in crystallinity while, for PBT, a substantial variation in the crystallinity degree happens more gradually and for cooling rates more than one order of magnitude larger. The experimental data obtained for PET and PBT in the two extreme cooling conditions are perfectly in line with the literature evidence. As regards the 3D-printed samples, the X-ray spectra of blends in Figure 9a show widened peaks for concentrations lower than 70% of PET and these peaks become less defined by increasing the PET content from 20% to 50%. Moreover, beyond 50% of PET, the 3D-printed samples made of 70% and 80% of PET are prevalently amorphous. This result suggests that, at the cooling condition of the 3D-printing process by increasing the PET content, the degree of crystallinity lowers, as already demonstrated from the DSC analysis. Furthermore, the 3D-printed neat PET is also completely amorphous. Reporting the degree of crystallinity calculated for printed samples on the graph in Figure 9b (stars), the obtained values are quite close to the lines obtained by the mixing rule, especially for the samples 20% PET, 30% PET, and 50% PET. For the 3D-printed blends at 70% and 80% of PET, the degree of crystallinity is much lower than the values predicted by the mixing rule. However, the strong lowering of the crystallinity degree of PBT/PET blends for a PET concentration higher than 50% during the cooling phase of the FFF process allows us to improve the printability of PBT, reducing the cost of the final 3D-printed artifact (due to the lower cost of PET) without making the printing condition and the printer setup more complicated (e.g., printing in controlled temperature atmosphere, using brim, adding filler, etc.) [14].

### 3.3. Mechanical Characterization of the Hollow Box-Shaped Specimens

**Figure 10:**
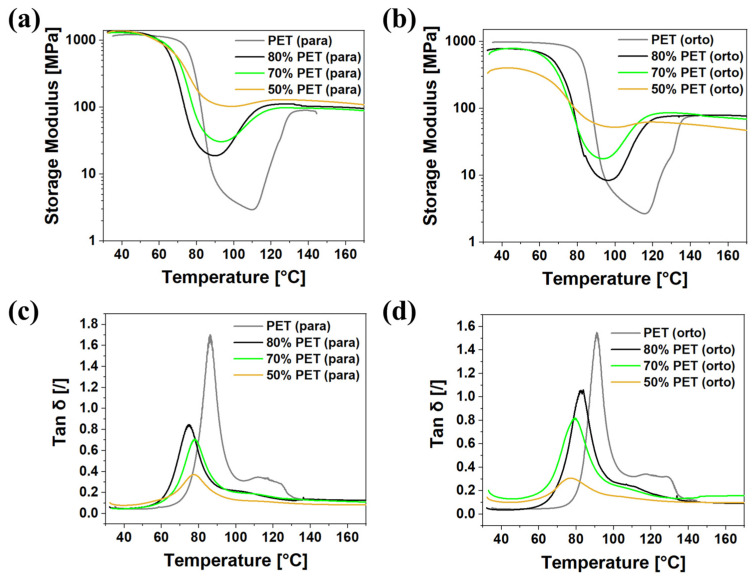
DMA analysis results: (**a**) elastic modulus in the filament direction (0°); (**b**) elastic modulus in the direction orthogonal to the printed filaments (90°); (**c**) loss tangent (tan δ) in the filament direction (0°); and (**d**) loss tangent (tan δ) in the direction orthogonal to the printed filaments (90°).

Figure 10a,b show the elastic modulus measured by DMA in both the filament direction (0°) and the orthogonal direction (90°), respectively. As expected, the samples exhibit superior performance in the filament direction. At lower temperatures (below 50 °C), the blend composition has a limited effect on the elastic modulus. However, as the temperature increases, differences between the samples become more pronounced. The PET sample shows a rapid reduction in the elastic modulus, reaching a minimum at about 110 °C, after which the modulus increases again due to the recrystallization of the amorphous phase. Other blends show a similar trend, with the reduction in elastic modulus proportional to the PET content.

Interestingly, the glass transition temperature (T_g_), determined from the peaks in the loss tangent (tan δ) spectrum (Figure 10c,d), does not follow a monotonic trend with the changing PET content. The Tg is the highest for the pure PET sample, decreases to the minimum for the 80% PET blend, and then increases again with decreasing PET content. This behavior is due to the combined effects of the degree of crystallinity in the printed samples and the Tg values of the individual components.

In the orthogonal direction, the blend composition significantly affects the modulus, even at lower temperatures. As the PBT content increases, the modulus decreases sharply. This is because layer adhesion, which is crucial for sample strength in this direction, is compromised by a higher PBT content. An increased PBT content accelerates crystallization, leading to greater warpage and instability. At higher temperatures, after complete recrystallization, all blends exhibit similar modulus values. This indicates that, at these temperatures, interlayer adhesion is complete, and all internal stresses are fully relaxed.

## 4. Conclusions

Comprehensive analyses of PBT/PET blends using FT-IR, TGA, X-ray diffraction, DSC, and mechanical testing have provided valuable insights into the effects of PET addition on the properties of PBT. Blend composition optimization enhances the printability and dimensional stability of the Poly(polyethylene terephthalate)/Poly(butylene terephthalate) blends.

The FT-IR analysis revealed that the PBT and PET polymers exhibit distinct spectral features despite their similar chemical structures. The absence of significant peak shifts or new peaks in the blend FT-IR spectra indicates that no substantial chemical interactions, such as transesterification, occurred during the extrusion process, which aligns with the TGA findings.

Thermogravimetric analysis (TGA) demonstrated that PET possesses better thermal stability than PBT, with degradation temperatures for PET being higher than those for PBT. The study confirmed that the blends do not undergo transesterification reactions, which supports the FT-IR findings. The increase in residue with a higher PET content reflects the higher char formation associated with PET, while PBT’s lower residue indicates its lesser char-forming tendency.

The X-ray diffraction (XRD) analysis showed that PBT and PET crystallize separately within the blends, forming distinct crystalline phases rather than cocrystals. The degree of crystallinity decreased with increasing PET content, particularly beyond 50%. The XRD results also indicated that PET reduces the crystallization rate of PBT, leading to a more amorphous structure in blends with a higher PET content. This observation was consistent with the DSC findings.

The differential scanning calorimetry (DSC) analysis revealed that the addition of PET to PBT leads to an increase in the glass transition temperature (T_g_) of the blends. The presence of cold crystallization peaks in the DSC curves suggests that a higher PET content slows down the crystallization rate of PBT during the cooling phase of 3D printing. This phenomenon explains the observed reduction in crystallinity and the improved dimensional stability of the printed parts.

Mechanical testing showed that, while the elastic modulus remained relatively stable across different blends, the PET content significantly influenced the strain at break. Blends with a higher PET content exhibited increased the rigidity and lower strain at break, indicating enhanced chain interactions and physical cross-linking. Notably, reducing crystallinity with an increasing PET content improved printability and reduced warpage, demonstrating the practical benefits of blending PET with PBT in 3D-printing applications.

In summary, the study confirms that PET effectively modifies the crystallization behavior and mechanical properties of PBT, leading to the enhanced dimensional stability and printability of the final 3D-printed artifacts. The findings highlight the potential for optimizing blend compositions to achieve the desired material properties for specific applications.

## Figures and Tables

**Table 1:** 3D-printing parameters.

Parameters	Specifics
Nozzle diameter	0.4 mm
Layer thickness	0.2 mm
Infill density	100%
Platform temperature	80 °C
Extrusion temperature	250 °C
Printing speed	80 mm/s

**Table 2:** Results of thermogravimetric analysis.

Sample	T_max_ [°C]	T_d_ [°C]	R [%]
PBT	394	359	4.55
20%PET	397	361	6.08
30% PET	400	361	6.11
50% PET	419	377	8.38
70% PET	422	382	9.45
80% PET	427	382	10.0
PET	424	392	12.4

**Table 3:** DSC results on spooled filaments of experimented blends.

Sample	T_g_ [°C]	T_c_ [°C]	∆H_m_ [J/g] I Heating	∆H_m_ [J/g] II Heating	∆H_c_ [J/g] Cooling
PBT	49.06	180.6	50.76	46.51	51.73
20%PET	56.91	169.1	54.77	39.48	44.12
30%PET	54.28	174.9	58.01	40.02	46.36
50%PET	61.79	148.0	46.68	30.53	35.45
70%PET	66.92	166.9	44.48	27.16	33.07
80%PET	74.28	180.4	45.78	35.18	39.71
PET	80.61	164.3	60.00	47.61	25.82

## Data Availability

The data presented in this study are available upon request from the corresponding author due to privacy reasons.

## Supplementary Materials

The following supporting information can be downloaded at: https://www.mdpi.com/article/10.3390/polym17020156/s1, Figure S1: (a) ^13^C NMR spectrum of 50%PET blend, (b) scheme of quaternary aromatic carbon in the case of copolymer (Z1 and Z2 positions), PET (X1 position), and PBT (X2 position) and the carbon atom in the CH2 group of PET (a position) and PBT (b position), Figure S2: DSC curves of first heating scan of PBT/PET blends in the form of spooled filament before printing (solid lines) and of 3D printed PBT/PET blends (dotted lines).
