# Supplementary material for: Effect of Crystallinity on the Printability of Poly(ethylene Terephthalate)/Poly(butylene Terephthalate) Blends
Source: Polymers (Basel). 2025 Jan 9;17(2):156. doi: 10.3390/polym17020156 (PMC11768295; doi:10.3390/polym17020156)
Supplement: Supplementary file 1 [file polymers-17-00156-s001.zip › polymers-3355326-supplementary.pdf]

Supplementary Materials

Effect of Crystallinity on the Printability of Poly(ethylene terephthalate)/Poly(butylene terephthalate) Blends

Francesca Aliberti [1,*], Maria Oliviero [2], Raffaele Longo [1], Liberata Guadagno [1,*] and Andrea Sorrentino [3]

[1] Department of Industrial Engineering, University of Salerno, Via Giovanni Paolo II, 132, 84084 Fisciano, Italy; rlongo@unisa.it
[2] Institute of Polymers, Composites and Biomaterials, National Research Council, P.le E. Fermi,1, 80055 Portici, Italy; maria.oliviero@cnr.it
[3] Institute of Polymers, Composites and Biomaterials, National Research Council, via Previati n.1/E, 23900 Lecco, Italy; andrea.sorrentino@cnr.it
* Correspondence: faliberti@unisa.it (F.A.); lguadagno@unisa.it (L.G.)

S1. NMR spectroscopy

The NMR spectra were recorded on a Bruker AM250 and a Bruker Avance 400 operating at 250 and 400 MHz for proton and 62.5 MHz and 100 MHz for carbon spectra, respectively. NMR samples were prepared by dissolving 10 mg of compounds in 0.2 ml of 1,1,1,3,3,3-Hexafluoro-2-propanol (Sigma-Aldrich) and 0.3 ml of $CDCl_3$ (Sigma-Aldrich).

NMR spectroscopy and FT-IR analysis were performed to examine the possible chemical interactions between PBT and PET that occurred during the extrusion blending phase or during the passage through the heated printing nozzle. Several studies explored the possibility of transesterification reactions in polyester blends like PET and PBT under specific processing conditions or heat treatment [38-42]. The absence of copolymers due to possible transesterification reactions has been verified via NMR spectroscopy on the printed samples. **Figure S1a** shows the NMR spectrum of the 50% PET blend, whose processing conditions are the same as all the other prepared blends. The carbon atoms investigated via NMR, characteristic of PET, PBT, and copolymers, are schematized in **Figure S1b**.

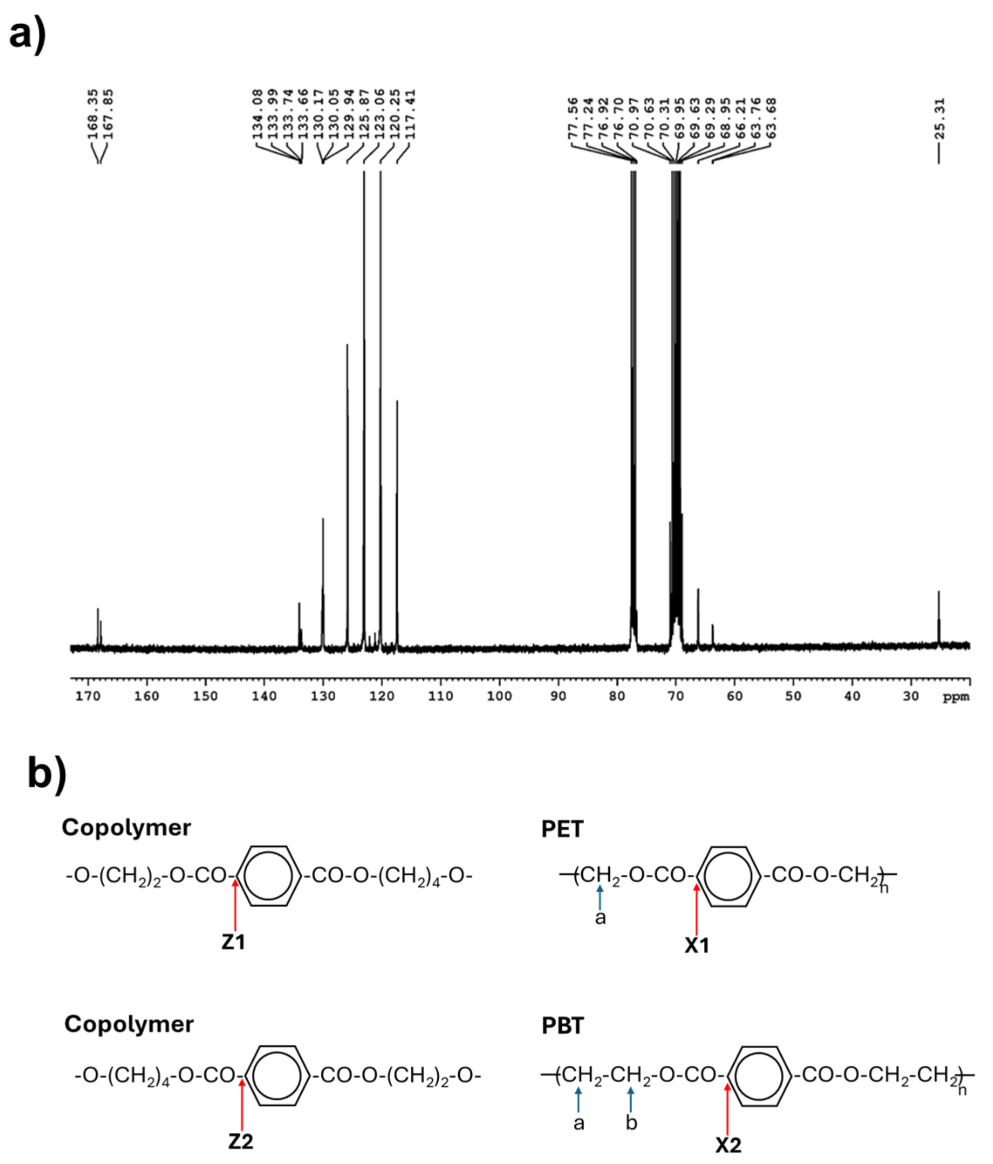

Figure S1. a) ^{13}C NMR spectrum of 50%PET blend, b) scheme of quaternary aromatic carbon in the case of copolymer (Z1 and Z2 positions), PET (X1 position), and PBT (X2 position) and the carbon atom in the CH_2 group of PET (*a* position) and PBT (*b* position).

Only two peaks are detected in the quaternary aromatic carbon region at 133.74 ppm and 134.08 ppm. The distance between the position of these two peaks is similar to that between the characteristic peaks of the quaternary aromatic carbon at positions X1 and X2 of PET and PBT, respectively [38], (see **Figure S1b**). If transesterification had occurred, there would also have been peaks due to hetero-sequences that should have the same intensity. From the integration of these peaks, it emerges that the area of the peak of 134.08 ppm is higher than that of the peak of 133.74 ppm because their areas give us the molar ratio between the two polymers. This relationship is confirmed by other characteristic peaks of PET and PBT at 66.21 ppm, attributed to the C of the CH_2 group in position a, and 68.95 ppm, attributed to the C of the CH_2 group in position b [53], as shown in **Figure S1b**. Overall, the peaks at 133.74 ppm (C in position X1) and 66.21 ppm (C of CH_2 in position a) are attributed to PET, while the peaks at 134.08 ppm (C in position X2) and 68.95 ppm (C of CH_2 in position b) refer to PBT. However, no peaks attributable to the quaternary aromatic carbon in the Z1 and Z2 positions of the copolymers are present in the ^{13}C NMR spectrum.

S2. DSC results on spooled filaments

In this section, the differences emerging from the comparison between the first and second heating scans of PBT, PET, and PBT/PET blends spooled filaments shown in Figure 4a are extensively discussed.

Comparing the melting peak of the PET alone in the first and second heating runs of Figure 4a, it is seen that the thermal history to which the polymer is subjected determines a profound rearrangement of the structural and morphological organization. The melting enthalpy in the first heating is 58 J/g, while in the second one, it is 34 J/g. In the second heating, the melting peak, in addition to having an intensity decrease of 41%, indicating a relevant decrease in the crystalline fraction, also shows a broader distribution of the crystallite dimensions (the melting peak opens at a lower temperature and closes at a higher temperature) compared to melting peak related to the first heating run.

Furthermore, comparing the melting peaks of the PET alone and the PET in the blend (80% PET) in the first heating run, it is seen that, together with the thermal history, also the presence of PBT affects the structural and morphological organization of the PET phase in the blend. The value of the melting enthalpy of the PET (peak centered at 252°C) in the first heating is 47 J/g (this value is normalized to the percentage of PET in the blend). This behavior evidences a decrease in the crystalline phase of the PET component, indicating that the PBT strongly hinders the PET crystallization already in the first heating run. This behavior is also manifested in the other analyzed blends. For example, the melting enthalpies of the PET in the blends of 70% PET and 30% PET are 41 J/g and 25 J/g, respectively, confirming the trend observed for PET alone and 80% PET samples. It is very likely that at the high heating and cooling rate (10°C per minute) used for the DSC curves, in the second heating run of 20% PET and 30% PET blends, the PET, in low percentage and with a reduced degree of crystallinity, is not able to crystallize. This justifies the absence of the PET melting peak in these blends, where only the melting peak of the PBT component is observed. On the contrary, when the percentage of PET is very high, as for 80% PET and 70% PET samples, the crystallization of PBT is hindered by the PET phase, and hence, only the melting peak of the PET is observed.

By comparing the first heating runs of the blends, we can also observe that the PBT component in the blend not only reduces the crystalline fraction of PET but also influences the size of the PET crystallites. In fact, the crystalline fraction of PET in the blends melts at a higher temperature than PET alone. This is a clear indication of more perfect PET crystallites in the blends.

S3. Comparison of DSC first heating curves of spooled filaments and 3D-printed samples of PBT/PET blends

Figure S2 reports the DSC first heating curves of spooled filaments (from Figure 4a) and printed samples of PBT/PET blends (from Figure 8).

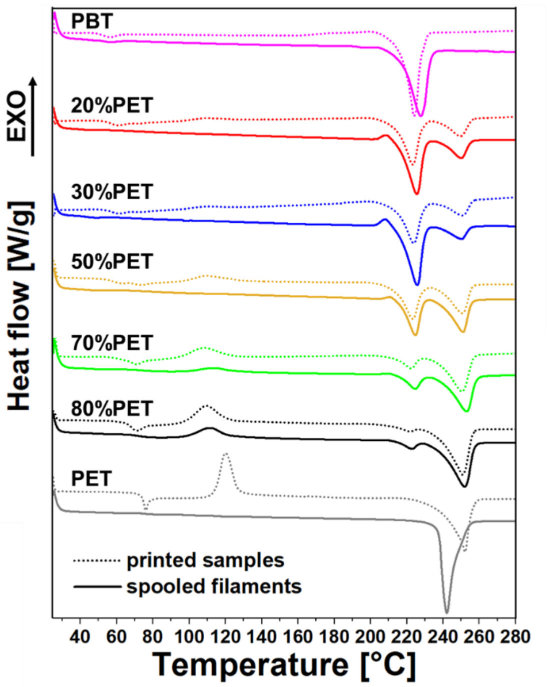

Figure S2. DSC curves of first heating scan of PBT/PET blends in the form of spooled filament before printing (solid lines) and of 3D printed PBT/PET blends (dotted lines).

From the comparison between DSC curves of spooled filaments (solid lines in **Figure S2**) and 3D-printed PBT/PET blends (dotted lines in **Figure S2**), it emerges that 3D-printed samples manifest a more visible glass transition temperature that increases by increasing the PET content in the blends following the same trend already found for spooled filaments reported in Figure 4c. Furthermore, the crystallization peak is more evident in the 3D-printed samples with respect to the spooled filaments. This means that the degree of crystallinity for the printed samples is lower than that of the respective spooled filaments. Although both spooled filaments and 3D-printed samples were cooled down in the air at room temperature, the different degrees of crystallinity could be due to the different dimensions of the spooled filaments (1.75 mm of diameter) with respect to thinner printed filaments (0.400 mm of diameter). The inner part of spooled filaments cools down slowly, having the time to crystallize. On the contrary, the printed filaments are so thin that they rapidly solidify in contact with the air at room temperature without the possibility of crystallizing. This also clarifies why PET, whose crystallization kinetics is slower than PBT, is completely crystalline in spooled filament while remaining with a higher percentage of amorphous phase after the printing process (an evident crystallization peak appears in the dotted line of PET in **Figure S2**). However, the DSC curves of 80%PET and 70%PET also show a crystallization peak for the spooled filaments. This behavior is most likely due to the presence of PBT, which partially obstacles the crystallization in the spooled filaments. Moreover, no differences in the melting peaks are detected for all the prepared blends before (solid lines of spooled filaments in **Figure S2**) and after printing (dotted lines of printed samples in **Figure S2**), suggesting that the additional thermal treatment due to the passage through the heated nozzle does not change the thermal properties of the material and confirming that transesterification did not occur as proven by NMR results.
